# Morphology-based radiomics signature: a novel determinant to identify multiple intracranial aneurysms rupture

**DOI:** 10.18632/aging.203001

**Published:** 2021-05-10

**Authors:** Xin Tong, Xin Feng, Fei Peng, Hao Niu, Baorui Zhang, Fei Yuan, Weitao Jin, Zhongxue Wu, Yuanli Zhao, Aihua Liu, Daming Wang

**Affiliations:** 1 Beijing Neurosurgical Institute, Capital Medical University, Beijing, China; 2 Department of Interventional Neuroradiology, Beijing Tiantan Hospital, Capital Medical University, Beijing, China; 3 Department of Neurosurgery, Beijing Hospital, National Center of Gerontology, Graduate School of Peking Union Medical College, Beijing, China; 4 Department of Neurosurgery, Peking University International Hospital, Beijing, China

**Keywords:** intracranial aneurysm, risk prediction, radiomics signature, radiomics features, nomogram

## Abstract

We aimed to develop and validate a morphology-based radiomics signature nomogram for assessing the risk of intracranial aneurysm (IA) rupture. A total of 254 aneurysms in 105 patients with subarachnoid hemorrhage and multiple intracranial aneurysms from three centers were retrospectively reviewed and randomly divided into the derivation and validation cohorts. Radiomics morphological features were automatically extracted from digital subtraction angiography and selected by the least absolute shrinkage and selection operator algorithm to develop a radiomics signature. A radiomics signature-based nomogram was developed by incorporating the signature and traditional morphological features. The performance of calibration, discrimination, and clinical usefulness of the nomogram was assessed. Ten radiomics morphological features were selected to build the radiomics signature model, which showed better discrimination with an area under the curve (AUC) equal to 0.814 and 0.835 in the derivation and validation cohorts compared with 0.747 and 0.666 in the traditional model, which only include traditional morphological features. When radiomics signature and traditional morphological features were combined, the AUC increased to 0.842 and 0.849 in the derivation and validation cohorts, thus showing better performance in assessing aneurysm rupture risk. This novel model could be useful for decision-making and risk stratification for patients with IAs.

## INTRODUCTION

Multiple intracranial aneurysms (MIAs) are encountered in approximately 20.1% of patients harboring unruptured IAs [1]. When a patient with unruptured MIAs is detected, determining which of the aneurysm is most likely to rupture is critical for determining the treatment strategy. For patients with both aneurysmal subarachnoid hemorrhage (aSAH) and MIAs, correct identification of the ruptured IA is also critical for planning treatment. After the IAST (International Subarachnoid Aneurysm Trial) research outcomes were reported, endovascular treatment has become the treatment choice for most ruptured IAs [2]. Therefore, rupture discrimination of MIAs is increasingly important because unlike in microsurgery, one cannot visually confirm the rupture in endovascular treatment.

Morphological parameters such as size [3–5], aspect ratio (AR) [6–9], size ratio (SR) [10–12], irregular shape [6–8], and flow angles [13, 14] have been suggested to be significant risk factors for IA rupture. However, studies on these morphological characteristics have reported conflicting results. This can be explained by the fact that these parameters are usually manually measured using different imaging techniques and measurement methodologies, and could differ among evaluators. In this study, we introduced objective and quantitative morphological features extracted from radiomics.

As an emerging branch of artificial intelligence, radiomics has been used to automatically extract a large number of objective and quantitative imaging features to select those most significantly associated clinical, pathological, molecular, and genetic characteristics so as to improve the diagnostic and prognostic accuracy and the evaluation of therapeutic efficacy [15–17]. There are only a few reports related to the application of radiomics-extracted morphological features in the detection of IAs and the stability of assessing small IAs [18, 19]. To the best of our knowledge, there are no reports illustrating whether a morphology-based radiomics signature will enable the superior assessment of the risks of IA rupture.

In this study, a subgroup of the patients from 3 centers with MIAs who developed aSAH were analyzed to characterize radiomics-extracted and traditional morphological features of the ruptured IA relative to other concomitant unruptured IAs. This was done to avoid confounding patient-specific characteristics [6, 7, 20]. We then developed and validated a combined nomogram that incorporates both the radiomics signature and traditional morphological features for the assessment of the individual risk of IA rupture in patients with MIAs.

## MATERIALS AND METHODS

### Ethical statement

This study was approved by the institutional research ethics boards of each center and conducted in accordance with the ethical standards and according to the 1964 Declaration of Helsinki.

### Study population

**Figure 1:**
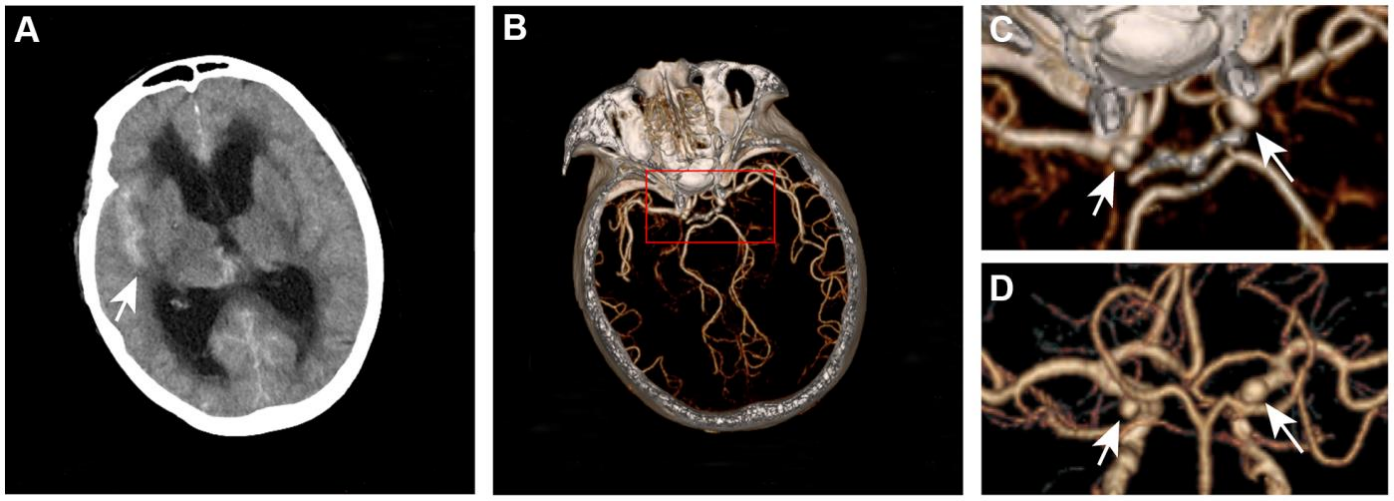
**Definitive hemorrhage pattern to confirm the ruptured aneurysm for patients who underwent endovascular or no treatment.** A 67-year-old woman presented with subarachnoid hemorrhage (**A**) was found to have left and right internal carotid aneurysms (**B**–**D**). The ruptured aneurysm is the right internal carotid aneurysm.

We retrospectively collected images and medical records from a consecutive series of patients with both aSAH and MIA treated at 3 hospitals in China (Beijing Tiantan Hospital, Beijing Hospital, and Peking University International Hospital) from January 2016 to December 2018. The inclusion criteria were as follows: patients with the diagnosis of aSAH and having at least two saccular IAs; patients with 3D-DSA diagnosed by Siemens Artis Zee System (Siemens Healthcare, Erlangen, Germany); sufficient image quality for segmentation; and patients with available clinical information. The ruptured IA was confirmed through microscopic visual assessment for patients with craniotomy treatment or through a definitive hemorrhage pattern on CT for patients who underwent endovascular or no treatment [21] (Figure 1). Patients in whom the ruptured IA could not be confirmed and those with other cerebrovascular diseases were excluded. Patients with aneurysm that has un-clear neck from the parent vessel were also excluded. All aneurysms included were randomly divided into 2 cohorts: the derivation cohort (70% of the IAs) and the validation cohort (30% of the IAs).

### Aneurysm segmentation and radiomics morphological features extraction

**Figure 2:**
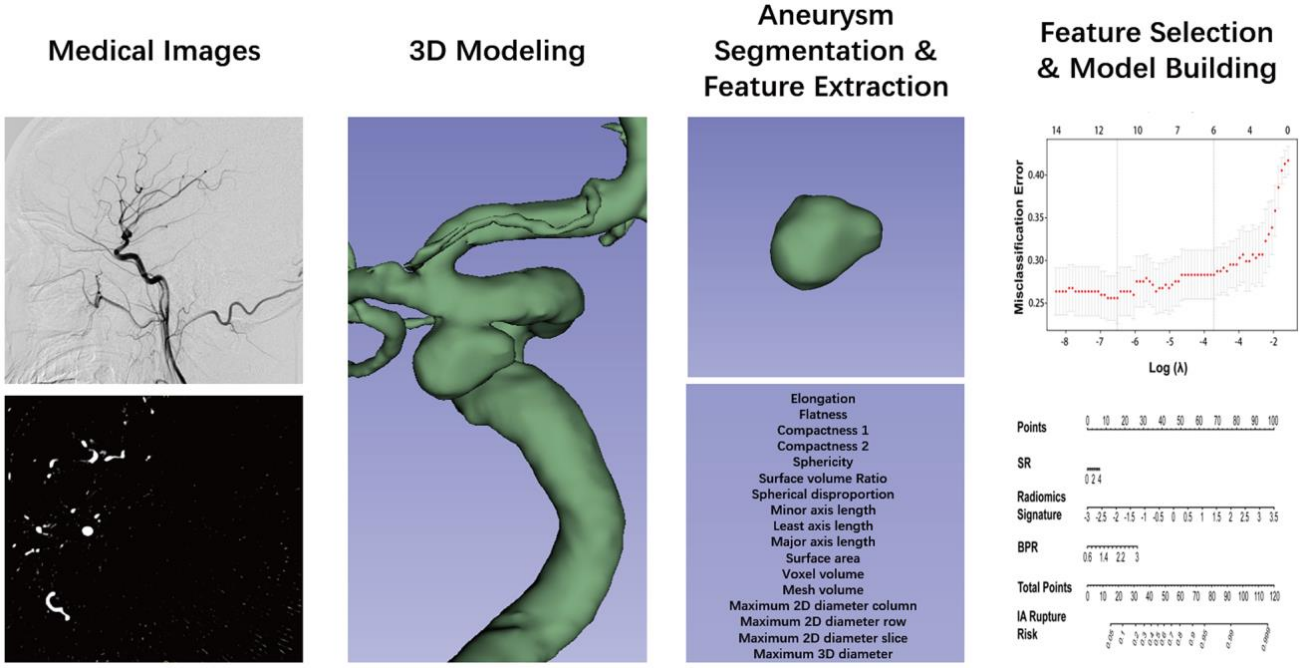
**Flow chart of the study.** The aneurysm was reconstructed from DSA images and using 3D slicer. The segmentation was performed by threshold and checked layer by layer. Then, the segmented label map and volume files were entered in the Pyradiomics package in the Python platform, and 17 radiomics morphological features were extracted for each aneurysm. The least absolute shrinkage and selection operator binary logistic were used to select the potential assessment factors and develop a radiomics signature. Along the radiomics morphological features, 16 traditional morphological features were combined and entered in the model construction analysis. Finally, the optimal model was performed in the nomogram.

The DSA image of each IA was reconstructed using an open-source software 3D Slicer [22] (version 4.9.0; http://www.slicer.org). In this study, the IA was defined as the region of interest (ROI). After reconstruction, IAs were manually segmented from the parent vessel at the neck of the IA [18, 19]. Two researchers (X.T. and X.F.) performed aneurysm segmentation. Disparities regarding the aneurysm reconstruction and segmentation between the researchers were further evaluated by 2 senior neurointerventionists each with 15 years of experience (AH.L. and DM.W.). The extraction of radiomics morphological features for each IA was completed with PyRadiomics in Python [16, 17, 19, 22–24], an open-source platform capable of extracting a large panel of morphological features from medical images. This radiomic quantification platform sets the reference standardization for feature definition and image processing [22]. In this study, 17 radiomics morphological features were extracted (Table 1). Detailed information on these features can be found at https://pyradiomics.readthedocs.io. A flow chart detailing the image processing operations performed in the present study is illustrated in Figure 2.

**Table 1:** Characteristics of the unruptured and ruptured IAs.

**Characteristic,** **mean±SD or Num(%)**	**Derivation cohort (n=176)**			**Validation cohort (n=78)**		
	**Unruptured IA** **(n=113)**	**Ruptured IA** **(n=63)**	***P***	**Unruptured IA** **(n=35)**	**Ruptured IA** **(n=43)**	***P***
**Traditional morphological features**						
Size (mm)	3.8±1.4	5.6±2.6	<0.001	4.2±1.8	5.5±2.1	0.007
Neck (mm)	3.4±1.0	3.8±1.3	0.020	3.6±1.7	3.9±1.2	0.357
Height (mm)	3.3±1.2	4.9±2.3	<0.001	3.4±1.3	4.8±1.9	<0.001
Width (mm)	3.4±1.1	4.4±1.8	<0.001	3.7±1.8	4.6±1.7	0.034
Diameter of parent artery (mm)	3.3±1.0	3.2±0.9	0.383	3.0±1.0	3.2±0.8	0.462
Branching to parent ratio	1.2±0.3	1.2±0.4	0.387	1.2±0.2	1.4±0.4	0.010
Neck to parent ratio	1.1±0.5	1.3±0.6	0.035	1.2±0.7	1.3±0.6	0.599
Aspect ratio	1.0±0.4	1.3±0.6	<0.001	1.1±0.5	1.3±0.6	0.132
Size ratio	1.0±0.5	1.5±0.8	<0.001	1.3±0.8	1.6±0.7	0.064
Width-Height ratio	1.1±0.4	1.0±0.4	0.155	1.1±0.3	1.0±0.3	0.353
Posterior circulation location	16(14.2)	12(19.0)	0.395	6(17.1)	9(20.9)	0.673
Irregular shape	33(29.2)	34(54.0)	0.001	10(28.6)	23(53.5)	0.027
Bifurcation aneurysm	35(31.0)	27(42.9)	0.114	11(31.4)	24(55.8)	0.031
Inflow angle (°)	105.5±24.7	110.8±27.6	0.190	110.3±26.9	109.7±29.1	0.927
Outflow angle (°)	97.7±26.9	92.9±27.6	0.266	102.6±29.2	96.5±25.8	0.329
Branching angle (°)	134.6±23.7	130.0±24.2	0.217	124.9±21.2	127.5±26.8	0.635
**Radiomics morphological features**						
Elongation	0.802±0.126	0.671±0.133	<0.001	0.811±0.112	0.717±0.118	0.001
Flatness	0.676±0.116	0.560±0.121	<0.001	0.690±0.113	0.589±0.107	<0.001
Compactness 1	0.039±0.003	0.036±0.004	<0.001	0.040±0.003	0.038±0.003	<0.001
Compactness 2	0.544±0.075	0.470±0.104	<0.001	0.571±0.076	0.506±0.078	<0.001
Sphericity	0.814±0.039	0.773±0.062	<0.001	0.828±0.039	0.794±0.043	0.001
Surface volume Ratio	2.069±0.580	1.867±0.628	0.033	2.009±0.633	1.743±0.592	0.060
Spherical disproportion	1.231±0.064	1.303±0.117	<0.001	1.210±0.060	1.263±0.072	0.001
Minor axis length	3.502±0.909	4.130±1.523	0.001	3.575±1.014	4.292±1.343	0.011
Least axis length	2.953±0.806	3.467±1.342	0.002	3.055±0.954	3.572±1.316	0.056
Major axis length	4.478±1.418	6.390±2.586	<0.001	4.539±1.783	6.145±2.183	0.001
Surface area	61.371±37.292	103.555±82.469	<0.001	65.908±48.541	104.050±71.951	0.009
Voxel volume	37.540±36.009	80.367±100.824	<0.001	44.097±51.718	82.383±83.145	0.020
Mesh volume	36.675±35.815	79.223±100.414	<0.001	43.417±51.353	81.385±82.954	0.021
Maximum 2D diameter column	4.792±1.429	6.233±2.292	<0.001	4.806±1.623	6.257±2.157	0.002
Maximum 2D diameter row	4.759±1.358	6.245±2.726	<0.001	4.976±1.590	6.194±2.369	0.011
Maximum 2D diameter slice	4.920±1.482	6.301±2.225	<0.001	4.894±1.532	6.365±2.391	0.002
Maximum 3D diameter	5.394±1.631	7.843±4.553	<0.001	5.458±1.958	7.371±2.700	0.001
**Radiomics signature**						
Rad-score	-1.020±1.118	0.730±2.004	<0.001	-1.123±1.149	0.327±1.245	<0.001

### Traditional morphological data acquisition

**Figure 3:**
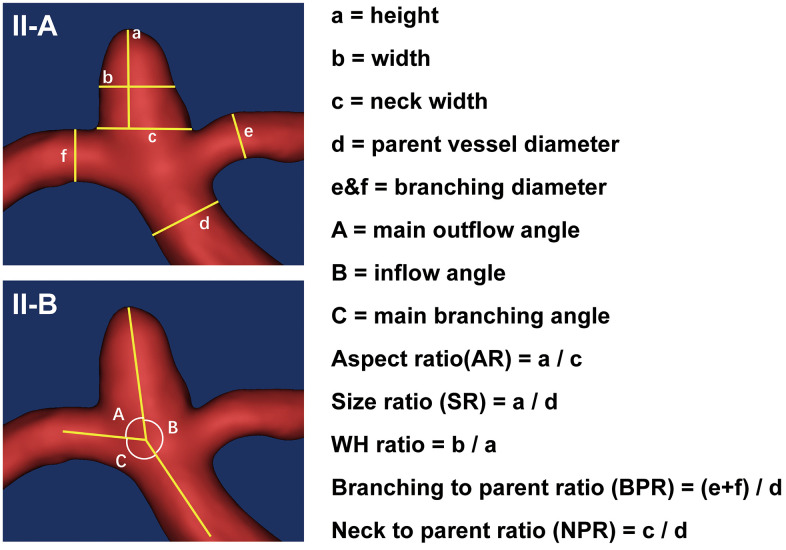
**Measurements of the traditional morphological features.** (**II-A**) schematically shows the aneurysm size and diameters of parent and branch vessels measurements, with the height (a), width (b), neck width (c), the diameter of the parent vessel (d), diameters of the branching vessel (e and f). (**II-B**) shows the angle measurements. The outflow angle (**A**) was defined as the angle at which the aneurysm flows outward to the distal parent artery in the sidewall aneurysm or to the daughter branch most approaching 180° in the bifurcation aneurysm. The inflow angle (**B**) was defined as the angle from the parent artery into the aneurysm. The main branching angle (**C**) was defined as the angle of the parent artery in the sidewall aneurysm or the angle between the parent artery and the daughter branch most approaching 180° in bifurcation aneurysm. In addition, several indicators were calculated: aspect ratio (AR) was defined as the ratio of aneurysm height (a) to the neck width (c); size ratio (SR) was defined as the ratio of aneurysm height (a) to the parent vessel diameter (d); WH ratio was defined as the ratio of aneurysm width (b) to the height (a); branching to parent ratio (BPR) was defined as the ratio of the sum of the diameters of the branch vessels (e + f) to the diameter of the parent artery (d) (in case of a sidewall aneurysm, the BPR was set to 1); neck to parent ratio (NPR) was defined as the ratio of the aneurysm neck width (c) to the parent artery diameter (d).

Traditional morphological features recorded from the angiograms included the IA number, location, size, neck, weight, inflow angle, outflow angle, branching angle, and diameters of the parent and branch vessels (Figure 3). Aneurysms with daughter sacs, multiple lobes, or other types of wall protrusions were defined as irregular. NPR, BPR, AR, SR, and WH ratio were calculated (Figure 3). All angiograms of IAs were re-evaluated and measured by two authors (X.F. and X.T.) and confirmed by two senior neurointerventionists (AH.L. and DM.W.).

### Statistical analysis

The continuous variables of patients’ baseline characteristics are presented as mean ± SD, and categorical variables are presented as percentages. All analyses were performed with IBM SPSS Statistics for Windows, version 25.0 (IBM Corp., Armonk, N.Y., USA) and R software (R Foundation for Statistical Computing, Vienna, Austria.). The major R software packages used in this study are listed in Supplementary Table 1. Statistical significance was set at *p <* 0.05.

### Feature selection and morphology-based radiomics signature construction

The assessment analysis included 16 traditional morphological features and 17 radiomics morphological features. Each traditional morphological feature with a probability value < 0.20 in the univariate analysis was included in the multivariate logistic regression analysis.

We used the LASSO algorithm to select the most discriminative radiomics morphological features from the derivation cohort. A rad-score was constructed for each IA from a linear combination of selected features that were weighted based on their respective LASSO coefficients (including both coefficient <0 and >0). The potential association of this morphology-based radiomics signature with IA rupture was assessed in the derivation and validation cohort by using a Mann-Whitney U test. Diagnostic validation was also performed in the derivation and the validation cohort.

### Development of aneurysm rupture assessment models

In this study, we constructed 3 models by the multivariate logistic regression analysis: 1) the MRS model, which included the radiomics signature and traditional morphological features, 2) the MRF model, which included the radiomics and traditional morphological features, and 3) the MTF model, which included traditional morphological features only. VIFs (< 2 were considered insignificant) were determined to evaluate the collinearity of combinations of final variables in models.

### Nomogram model selection and performance assessment

The performance of the models was tested in the derivation and validation cohorts. We used AUCs to compare the discriminatory efficacy of the different models. Based on these analyses, the optimal model was developed into the nomogram. The discrimination accuracy and the calibration of the nomogram were assessed using AUCs, calibration curves, and the Hosmer-Lemeshow test. The Brier score, ranging from 0 (excellent discriminate ability) to 1 (worst discriminate ability), was used to determine the overall performance of each model.

### Clinical usefulness

DCA evaluates assessment models and visualizes the net benefit derived from the use of a specific assessment model. Thus, we conducted DCA to determine the clinical usefulness of the nomogram in this study.

## RESULTS

### Study population

A total of 254 IAs in 105 patients were included, including 148 unruptured IAs and 106 ruptured IAs (one patient had two ruptured IAs 12 months apart). Among the 105 patients, 75 (71.4%) were women. The median age of the patients was 58.5 years (range, 31 - 85 years). Current smoking and hypertension were present in 26 (24.8%) and 64 (61.9%) patients, respectively. (Supplementary Table 2) Ruptured IA was not the largest IA was found in 32 (30.5%) patients. In this study, 176 IAs were randomized into the derivation subset, and 78 IAs were randomized into the validation subset. Details of IAs in the derivation and validation subsets are shown in Table 1.

### Features selection and morphology-based radiomics signature construction

The comparison of traditional morphological and radiomics features between ruptured and unruptured aneurysms is summarized in Table 1. The eleven traditional morphological features in the derivation cohort with *p <* 0.20 that were analyzed using multivariable logistic regression included: size, neck, height, width, AR, SR, irregular shape, neck, branching to parent ratio (BPR), neck to parent ratio (NPR), and bifurcation.

**Figure 4:**
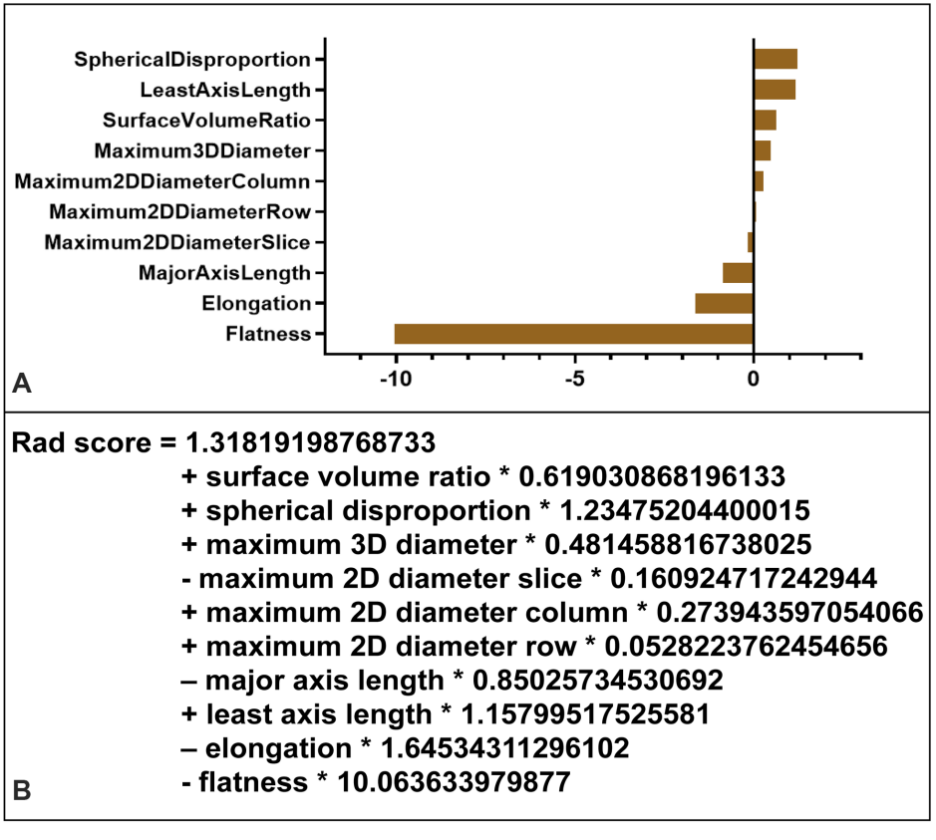
**Radiomics signature score (rad-score) calculation.** (**A**) Radiomic features ranked by coefficients of the least absolute shrinkage and selection operator (LASSO) binary logistic regression model. The flatness was the most correlated indicator with IA rupture. (**B**) Radiomics signature (rad-score) was constructed from a linear combination of selected features that were weighted based on their respective LASSO coefficients.

Ten radiomics morphological features were identified as potential indicators, including surface volume ratio, spherical disproportion, maximum 3D diameter, maximum 2D diameter slice, major axis length, least axis length, maximum 2D diameter column, maximum 2D diameter row, elongation, and flatness. These ten features had coefficients > 0 in the least absolute shrinkage and selection operator (LASSO) logistic regression model and were included in the radiomics signature score (rad-score) calculation formula (Figure 4).

### Performance of the morphology-based radiomics signature

**Figure 5:**
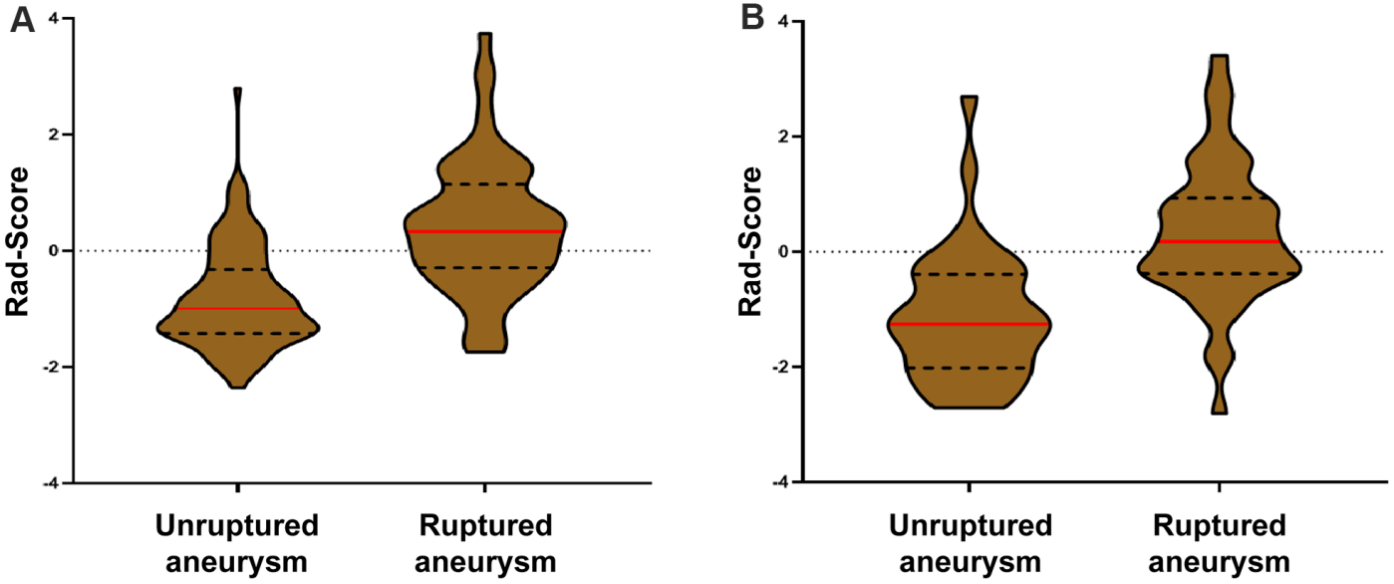
**Violin plots of the radiomics signature score (rad-score).** There was a significant difference in the rad-score between unruptured IA and ruptured IA in the derivation cohort (*p* < 0.001, III-**A**), which was then confirmed in the validation cohort (*p* < 0.001, III-**B**).

**Figure 6:**
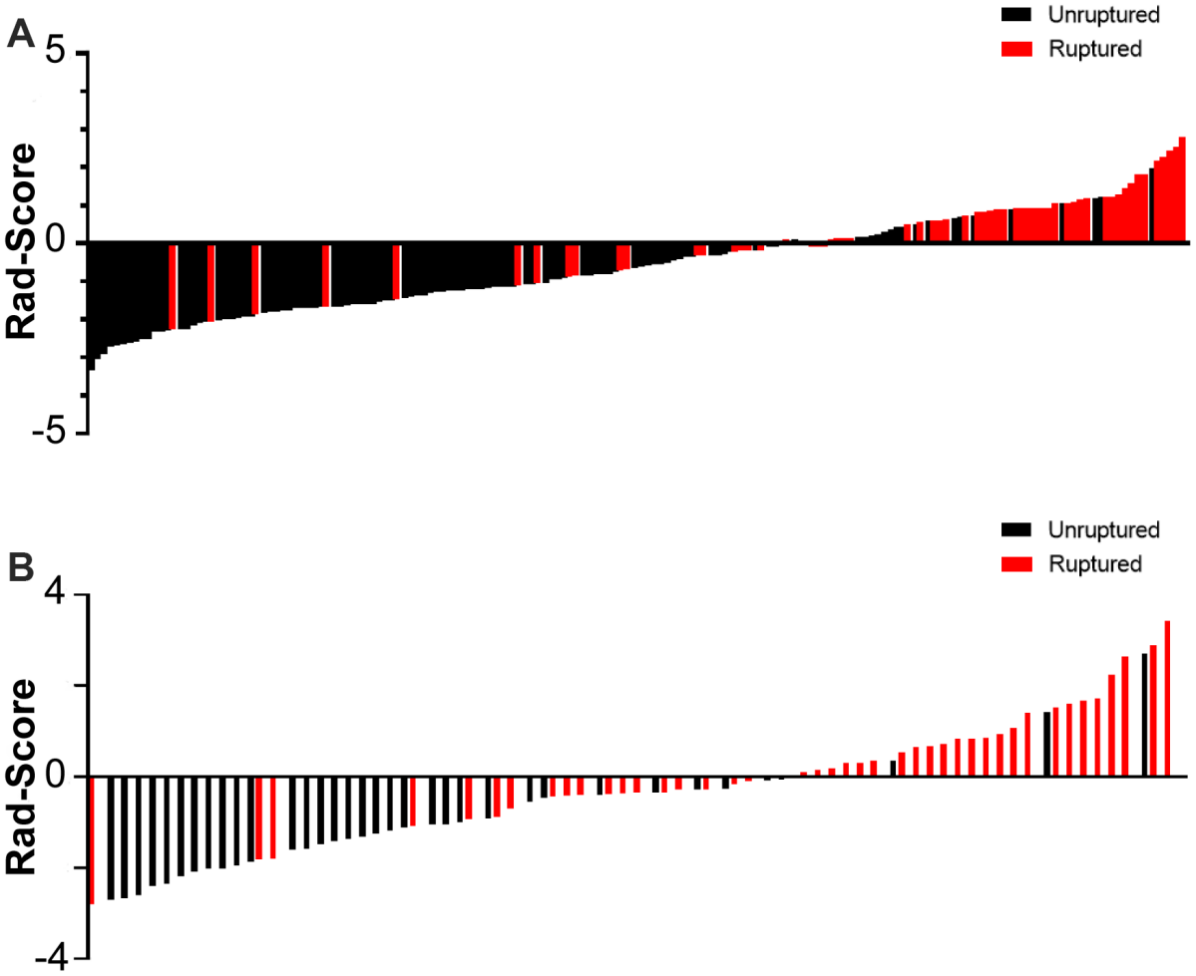
Rad-score for every aneurysm in each in the derivation (**A**) and validation cohort (**B**).

The rad-scores of the ruptured group were significantly higher than that of the unruptured group in both the derivation and validation cohorts (*p* < 0.001, Table 1, Figure 5). A significant association between the rad-score and IA rupture was also found in the IAs at a different location (Supplementary Table 3). The radiomics signature yielded areas under the curve (AUCs) of 0.814 (CI 95%, 0.746-0.881) in the derivation cohort and 0.835 (CI 95%, 0.739-0.930) in the validation cohort (Figure 6).

### Individualized nomogram model development and selection

Multivariate logistic regression analysis yielded 3 rupture discriminations models:

the morphology-based radiomics signature (MRS) model, which include radiomics signature and traditional morphological features,

odds _MRS_ = [0.90(Radiomics Signature) + 0.79(SR) + 1.23(BPR) – 2.84];

the morphology-based radiomics features (MRF) model, which include radiomics features and traditional morphological features,

odds _MRF_ = [0.26(Maximum 3D Diameter) +0.88 (SR) - 5.89(Flatness) + 0.31]; and

the morphology-based traditional features (MTF) model, which include only traditional morphological features,

odds _MTF_ = [1.66 (AR) + 0.96 (NPR) + 0.73 (Irregular shape) – 3.90];

where odds are the ratios of the probability of ruptured status to the probability of unruptured status of an IA.

Independently significant discriminants of the MRS model included radiomics signature, SR, and BPR. Discriminants of the MRF model included flatness, maximum 3D diameter, and SR. Discriminants of the MTF model include AR, NPR, and irregular shape (Table 2). We found that only SR was incorporated in both the MRS model (OR, 3.430; CI 95%, 1.275-4.521) and the MRF model (OR, 2.400; CI 95%, 1.226-4.900).

**Table 2:** Multivariate analysis for patient with aSAH and MIAs.

**Model**	**Risk factors**	**Odds ratio (95% CI)**	***P***	**VIF**
**MRS** **Model**	Radiomics Signature	2.466(1.756-3.618)	0.003	1.116
	SR	3.430(1.275-4.521)	0.023	1.073
	BPR	3.430(1.275-9.890)	0.018	1.056
**MRF** **Model**	Flatness	0.003(<0.001-0.0600)	<0.001	1.044
	Maximum 3D Diameter	1.359(0.109-16.488)	0.010	1.307
	SR	2.400(1.226-4.900)	0.013	1.295
**MTF** **Model**	AR	5.240(2.451-12.099)	<0.001	1.149
	NPR	2.621(1.373-5.257)	0.004	1.144
	Irregular shape	2.078(1.036-4.173)	0.039	1.022

MRS model, morphology-based radiomics signature model; MRF model, morphology-based radiomics features model; MTF model, morphology-based traditional features model; SR, size ratio; AR, aspect ratio; NPR, neck to parent diameter ratio; VIF, variation inflation factors.

**Figure 7:**
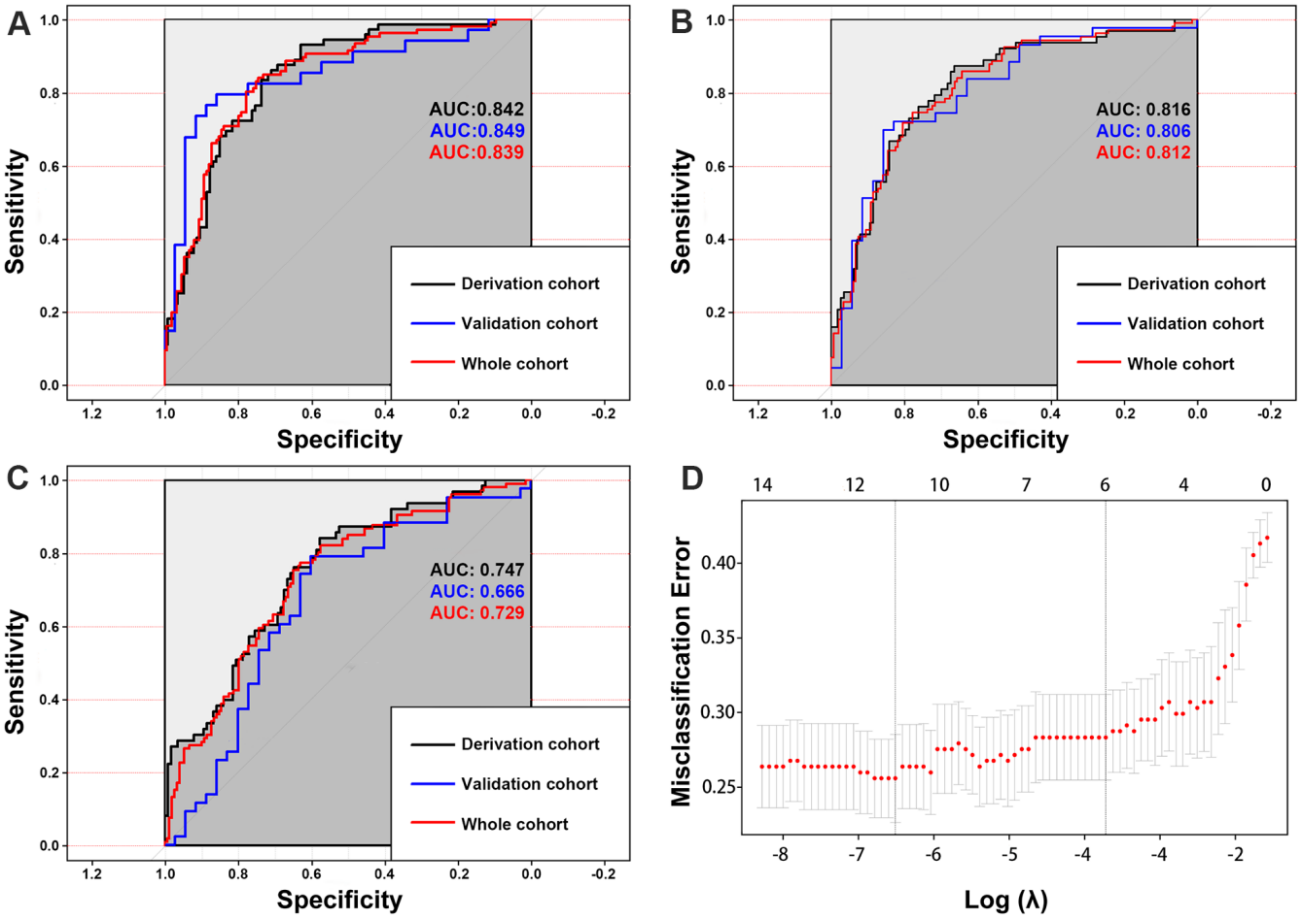
The area under the curves (AUCs) shows that the morphology-based radiomics signature model (**A**) has better discrimination compared with the morphology-based radiomics features model (**B**) and morphology-based radiomics features model (**C**). Radiomics morphological feature selection used the LASSO binary logistic regression model (**D**).

**Figure 8:**
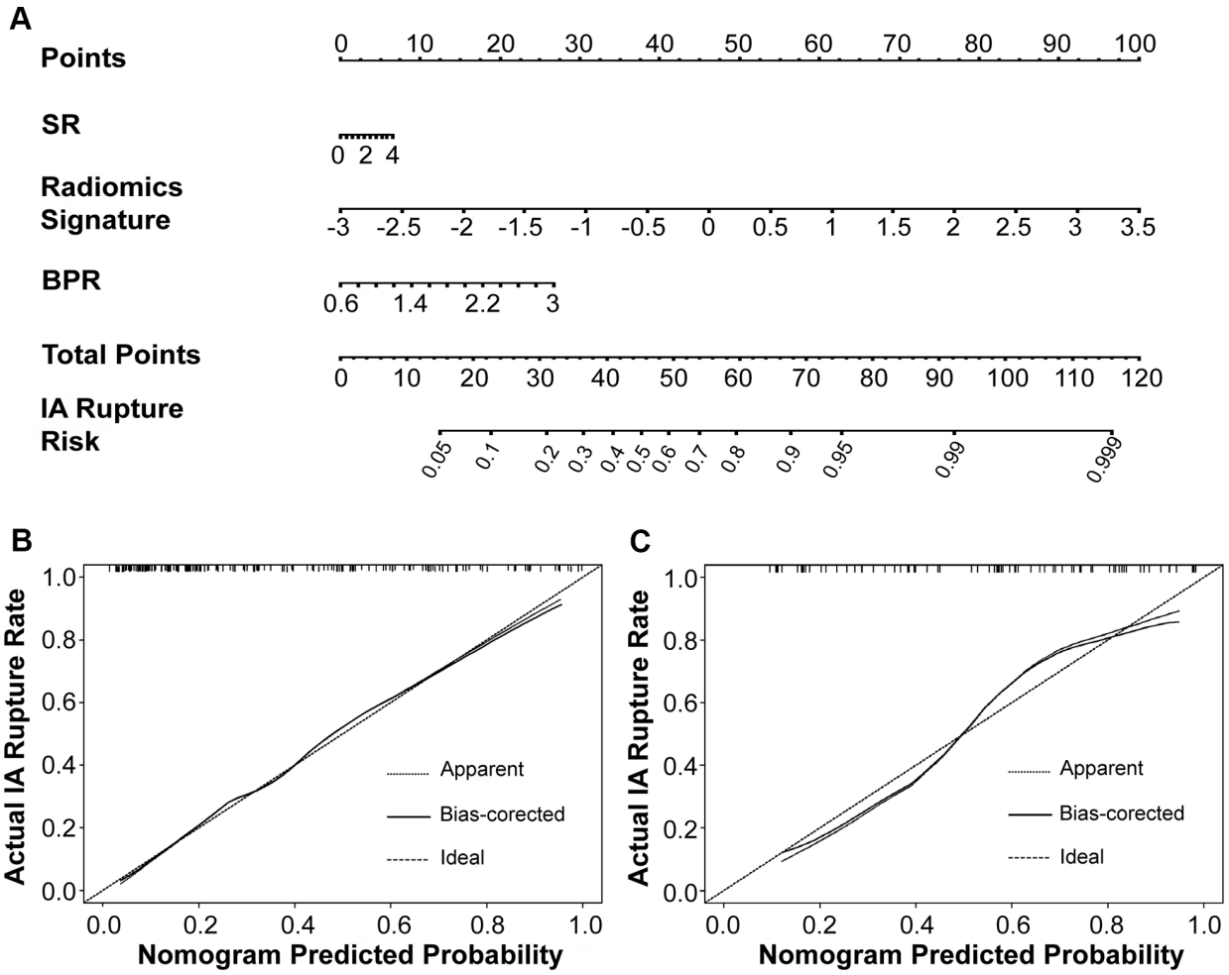
The morphology-based radiomics signature model was developed into nomogram (**A**). Calibration curves suggest that our nomogram performed well in both the derivation (**B**) and validation (**C**) cohorts.

For all three models, the variance inflation factors (VIFs) of all the candidate indicators ranged from 1.050–1.307, demonstrating that there was no collinearity in any of the three models. The MRS model (AUC, 0.842; 95% CIs, 0.786-0.899) assessed the risk of aneurysm rupture in the derivation cohort more accurately than the MTF model (AUC, 0.747; 95% CIs, 0.673-0.822) or the MRF model (AUC, 0.820; 95% CIs, 0.755-0.885) (Figure 7). In the validation cohort, the MRS model (AUC, 0.849, 95% CIs, 0.752-0.946) also had the largest AUC compared with the MTF (AUC, 0.666; 95% CIs, 0.539-0.793) and MRF models (AUC, 0.799; 95% CIs, 0.697-0.902). As the optimal model, the MRS model was developed as the nomogram (Figure 8A).

### Nomogram model validation and performance assessment

The AUC of 0.842 in the derivation cohort and 0.849 in the validation cohort showed good discrimination by the nomogram. Brier scores of 0.164 and 0.168 showed good overall performance of the MRS model in the derivation and validation cohorts, respectively (Table 3). The calibration curve of the nomogram demonstrated good agreement between estimating and observation results (Figure 8). The Hosmer-Lemeshow test of the nomogram in the derivation cohort (*p* = 0.782) showed no departure from perfect fit, which was confirmed in the validation cohort (*p* = 1.000).

**Table 3:** Evaluation of discrimination and calibration abilities of the models.

	**MRS model**			**MRF model**			**MTF model**		
	**Derivation cohort**	**Validation cohort**	**Total cohort**	**Derivation cohort**	**Validation cohort**	**Total cohort**	**Derivation cohort**	**Validation cohort**	**Total cohort**
Brier score	0.160	0.137	0.159	0.162	0.174	0.173	0.190	0.227	0.208
AUC (95% CI)	0.842 (0.786-0.899)	0.849 (0.752-0.946)	0.840 (0.790-0.890)	0.820 (0.755-0.885)	0.799 (0.697-0.902)	0.816 (0.764-0.869)	0.747 (0.673-0.822)	0.666 (0.539-0.793)	0.729 (0.667-0.791)
Hosmer-Lemeshow test (*p* value)	0.366	1.000	1.000	0.098	0.494	0.950	0.139	0.171	0.954

AUC, area under the receiver operating characteristic curve; MRS model, morphology-based radiomics signature model; MRF model, morphology-based radiomics features model; MTF model, morphology-based traditional features model.

### Clinical usefulness

**Figure 9:**
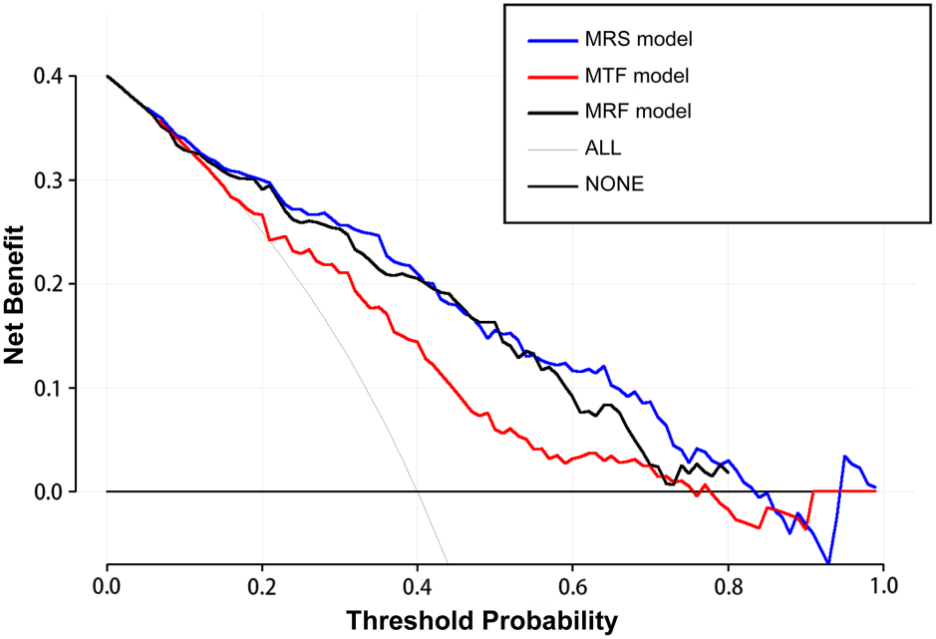
The decision curve analysis demonstrates the morphology-based radiomics signature model (MRS model) has a larger net benefit compared with the morphology-based radiomics features model (MRF model) and morphology-based traditional features model (MTF model) for the assessment of aneurysm rupture risk.

Compared to the MRF and MTF models, the MRS model showed larger net benefits in the decision curve analysis (DCA) (Figure 9). The DCA showed that if the probability of IA rupture generated by the MRS nomogram model is between 0.10–0.80, the use of nomogram to assess the IA rupture risk adds more benefit than either of the treat-all or the treat-none strategies (Figure 9).

## DISCUSSION

Our study has important implications for clinical practice. To our knowledge, this study was the first to attempt to establish a reliable morphology-based radiomics signature nomogram to assess the risk of IA rupture in MIA patients. This nomogram incorporated both radiomics signature and traditional morphological features and showed larger benefit gains in assessing the risk of IA rupture compared with models based on radiomics or traditional morphological features.

### Radiomics signature was significantly associated with MIA rupture

Previous studies have shown that IA size is the most important morphological risk factor for ruptures [3–5, 25]. However, other studies showed that in 12.5%–33% of patients with MIA, the ruptured IA was not the largest one [6, 7]. In our study, similarly, this was 30.5%. An irregular shape has also been reported as one of the most important factors for predicting IA rupture [6–8]. Some studies defined IA with multiple lobes, daughter sacs, or other types of wall protrusions as irregular [5–7, 26]. Such descriptions are qualitative and rely on the evaluator's experience with an ill-defined threshold. A few other studies used morphology indexes such as AR [6–9], SR [10–12], and flow angles [13, 14] for IA rupture risk assessment. However, these studies reported conflicting results. This may be because traditional morphological features were manually measured using different imaging techniques and measurement methodologies, which could differ among evaluators. Furthermore, compared with the radiomic morphological features automatically obtained based on artificial intelligence, traditional morphological features that are mainly measured in a 2-dimensional projection are not sufficient to judge the overall morphology of the IA. All these contradictions emphasize the need for a new tool to assess aneurysm rupture risk.

In this study, we introduced radiomics morphological features and developed a morphology-based radiomics signature nomogram model in patients with aSAH and MIA. We used LASSO regression to select potential indicators and combined them via a linear equation weighted by each indicator's respective coefficients to construct the radiomics signature. This method allowed us to incorporate individual radiomics morphological features into a feature panel in order to do multi-feature analyses and it has been used in several recent studies [17, 27, 28]. We found that the rad-scores of the ruptured group were significantly higher than that of the unruptured group in both derivation and validation sets (Figures 5, 6). More importantly, our result shows that the morphology-based radiomics signature performs well in assessing ruptured aneurysms in patients with SAH and MIAs, with an AUC of 0.814 in the derivation cohort and 0.835 in the validation cohort (Figure 6). After grouping by location, the radiomics signature still showed a better stratified ability for detecting IAs at different locations (Supplementary Table 3). Furthermore, the radiomics signature had better assessment accuracy than the MTF model based on traditional morphological features only (AUC, 0.814 vs. 0.747). These results confirm that the morphology-based radiomics signature, as a new indicator established by radiomics and machine learning, has great values in IA rupture risk assessment.

### Development and validation of morphology-based radiomics signature nomogram

Several methods, such as clinical risk factor assessments and scoring systems, have been used in IA stratification in clinical practice [4, 5, 29]. However, the usage of these clinical tools in clinical practice remains controversial [30–32], especially for patients with MIAs. This study intended to establish a relatively accurate, convenient, and noninvasive method for assessing IA rupture based on radiomics-extracted morphological features. Therefore, we established three different models and selected the optimal model to develop a nomogram. As a result, we found that the MRS model (AUC, 0.842), which incorporated radiomics signature and traditional morphological features, was more accurate in IA risk assessment than the MRF model (AUC, 0.820) and the MTF model (AUC, 0.747). The DCA also showed that the MRS model was associated with larger net benefits than the MRF and MTF models. Our study results with those mentioned above indicate that the combination of radiomics-extracted and clinical traditional morphological variables has a complementary and synergistic effect in assessing IA rupture in patients with MIA.

In this study, we found that all three models had some shared and some distinct characters. All models included features that reflect the shape of the IA, such as radiomics signature in the MRS model, flatness in the MRF model, as well as AR and irregular shape in the MTF model. An irregular shape has been reported as one of the most important factors for predicting IA rupture [6–8]. These geometries tend to harbor vortices, which may promote the infiltration of inflammatory cells [33]. Moreover, the local disturbed flow pattern coursed by the irregular shape of IAs may be more vulnerable to form thrombus, which could aggravate inflammatory cell infiltration and the proteolytic degradation of the IA wall [34–36]. In the MTF model, AR and irregular shape, as typical morphological features, have been widely studied and correlated with IA rupture [6–9]. In the MRF model, the flatness was a novel morphological radiomics index, which shows the relationship between the largest and smallest principal components in shape [17–19]. Liu et al. identified flatness as the most important determinant to predict small IA stability [19]. In our study, LASSO binary logistic regression analysis showed that flatness was also the most correlated indicator for IA rupture (Figure 4). Compared to AR, NPR, and irregular shape, flatness may be a more objective and reliable way to describe the IA morphology. In the MRS model, the radiomics signature reflected the overall morphology of the aneurysm more comprehensively than a single traditional or radiomics morphological feature. Interestingly, NPR was included in the MTF model, and SR was included in the MRS and MRF model. NPR is defined as the ratio of the neck width to the parent artery diameter, which is affected by the IA location and parent vessel. SR was found to be a more robust morphometric feature than AR or size, which can also be affected by the location and parent vessel of the IA [10–12]. In addition, maximum 3D diameter, which was included in the MRF model, represented the largest diameter of the IA in 3 dimensions and could be more accurate than traditional size measurements relying on manual measurement by the naked eye [17–19].

Previous studies have linked IA rupture and radiomics features. Liu et al. attempted to predict aneurysm stability with radiomics morphological features. However, 13.7% in their case group were growing or symptomatic unruptured aneurysm, so it was not suitable for identifying IA ruptured in MIA patients [19]. In addition to morphological features, more radiomic features could be extracted from CT or MR imaging. However, this is a technical challenge for IA because IA is a cystic vascular disease, unlike other substantial lesions such as tumors. In fact, as the gold standard for diagnosis and evaluation of IA [37]. 3D digital subtraction angiography (3D-DSA) reconstruction mainly reflects the morphological characteristics of the aneurysm and parent artery, which are very important factors affecting the risk of aneurysm rupture. Therefore, we only extracted morphological-based radiomics features from 3D-DSA. Further research will be conducted using on high-resolution magnetic resonance images to extract more imaging features (such as grayscale) other than the morphological factors.

### Strengths and limitations

Our study was specifically designed to yield accurate and reliable results. Radiomics is the mining of quantitative and objective image parameters and the evaluation of IA morphological characteristics in multiple dimensions. We included patients who had both ruptured and unruptured IAs. Similarly, three previous articles have also studied this subgroup of patients [7, 9, 38]. All IAs were exposed to the same internal milieu within this study population as patient-specific parameters and standardized by their own internal controls. This makes our model more accurate [7, 9, 38]. Furthermore, we used a nomogram to develop our IA rupture risk assessment tool. Nomograms have been widely accepted as reliable tools to determine quantitative risk factors for clinical events.

This study has some limitations. First, our study was based on post IA rupture imaging, which may not accurately reflect the original morphology of existing IAs. This shortcoming may be further magnified by the accuracy of the radiomic morphological feature extraction. Second, the retrospective design of our study may have introduced some bias, and we did not have an external validation. The number of cases in the verification group was relatively small (76 IAs). However, because only 2 or 3 variables were used in each model, this sample size was statistically satisfactory. Third, this research is based on semi-automatic segmentation procession, and manual segmentation of IA neck may introduce discrepancy. Fourth, incorporated both radiomics signature and traditional morphological features, our model does not fully cover the shortcomings of manual extraction of parameters, so further research needs to consider fully automatic extraction of all aneurysm morphological and radiomic parameters. Lastly, models in this study did not include factors such as hemodynamics [20], genetics [39], and wall enhancement [40]. The inclusion of these parameters may improve the assessment accuracy of the model but may also increase the complexity of the model and limit its clinical application [25].

## CONCLUSIONS

To the best of our knowledge, this study was the first attempt to establish a reliable morphology-based radiomics signature nomogram to assess the risk of IA rupture in MIA patients. This nomogram may provide a relatively accurate, convenient, and noninvasive method for the decision-making and risk stratification for patients with MIAs.

### Availability of data and material

Some or all data, models, or code generated or used during the study are available from the corresponding author by request.

### Consent for participation and publication

Informed consents for participation and publication were obtained from all participants.

## Supplementary Material

Supplementary Tables
