# Supplementary material for: Morphology-based radiomics signature: a novel determinant to identify multiple intracranial aneurysms rupture
Source: Aging (Albany NY). 2021 May 10;13(9):13195–210. doi: 10.18632/aging.203001 (PMC8148474; doi:10.18632/aging.203001)
Supplement: Supplementary Tables [file aging-13-203001-s001.pdf]

SUPPLEMENTARY TABLES

Supplementary Table 1. Major packages of R software used in this study.

Functions	R package
logistic regression analysis	glm
Plot the receiver operating curve (ROC) and measure the area under the ROC (AUC)	pROC
Hosmer and Lemeshow goodness of fit (GOF) test	ResourceSelection
Plot bar diagrams	ggplot2
Plot calibration curves, Brier score and develop nomogram	rms
Decision curve analysis (DCA)	rmda

Supplementary Table 2. Demographic and clinical features of the whole cohort.

	Whole cohort
Age	58.5 (31 - 85)
Female	75 (71.4%)
Hypertension	64 (61.9%)
Hyperlipidemia	12(11.4%)
Diabetes	11(10.5%)
Heart disease	13 (12.4)
Smoking	26 (24.8%)
Drinking	22 (21.0%)

Supplementary Table 3. Stratified analysis of the association between the morphology-based radiomics signature and aneurysm rupture in the whole population.

Locations	Radiomics signature		*P* - value
	Unruptured aneurysm	Ruptured aneurysm	
Internal carotid artery	-1.032(1.061)	0.737(2.080)	<0.001
Anterior cerebral artery	-1.433(0.807)	0.542 (1.560)	<0.001
Middle cerebral artery	-0.979(0.822)	0.451(1.208)	<0.001
Posterior circulation	-0.863(1.720)	0.239(1.221)	0.023
